# Applications of Dynamic Clamp to Cardiac Arrhythmia Research: Role in Drug Target Discovery and Safety Pharmacology Testing

**DOI:** 10.3389/fphys.2017.01099

**Published:** 2018-01-04

**Authors:** Francis A. Ortega, Eleonora Grandi, Trine Krogh-Madsen, David J. Christini

**Affiliations:** ^1^Physiology, Biophysics, and Systems Biology Graduate Program, Weill Cornell Graduate School of Medical Sciences, New York, NY, United States; ^2^Department of Pharmacology, University of California, Davis, Davis, CA, United States; ^3^Greenberg Division of Cardiology, Weill Cornell Medical College, New York, NY, United States

**Keywords:** dynamic clamp, cardiac electrophysiology, cardiac modeling, arrhythmia mechanisms, antiarrhythmic drugs, pharmacology & drug discovery

## Abstract

Dynamic clamp, a hybrid-computational-experimental technique that has been used to elucidate ionic mechanisms underlying cardiac electrophysiology, is emerging as a promising tool in the discovery of potential anti-arrhythmic targets and in pharmacological safety testing. Through the injection of computationally simulated conductances into isolated cardiomyocytes in a real-time continuous loop, dynamic clamp has greatly expanded the capabilities of patch clamp outside traditional static voltage and current protocols. Recent applications include fine manipulation of injected artificial conductances to identify promising drug targets in the prevention of arrhythmia and the direct testing of model-based hypotheses. Furthermore, dynamic clamp has been used to enhance existing experimental models by addressing their intrinsic limitations, which increased predictive power in identifying pro-arrhythmic pharmacological compounds. Here, we review the recent advances of the dynamic clamp technique in cardiac electrophysiology with a focus on its future role in the development of safety testing and discovery of anti-arrhythmic drugs.

## Introduction

The search for successful anti-arrhythmia therapeutics is rooted in the voltage clamp and current clamp techniques, which have provided the mechanistic details behind the ionic membrane currents that compose the cardiac action potential (AP). While basic science has made great leaps in identifying and characterizing the basic factors involved in arrhythmia, the translation of these advances into successful therapies has been lackluster. Nonetheless, investigators have been using a combination of experimental and computational approaches to unravel the complex mechanisms underlying cardiac arrhythmia. Using this approach, experimental measurements, typically in single cells from mammalian hearts, are used to develop biophysically detailed mathematical models that can be scaled up to the tissue and whole-organ levels where arrhythmia occurs. Unlike experiments, computational modeling readily allows for the precise perturbation of particular parameters individually or in controlled combinations (simulating, e.g., the multifactorial nature of many disorders), but results are reliant on the accuracy of the model and its many components. The dynamic clamp technique is a merger between experimental and computational techniques that has been gaining traction as a hybrid method for elucidating arrhythmia mechanisms and possible therapeutics.

**Figure 1:**
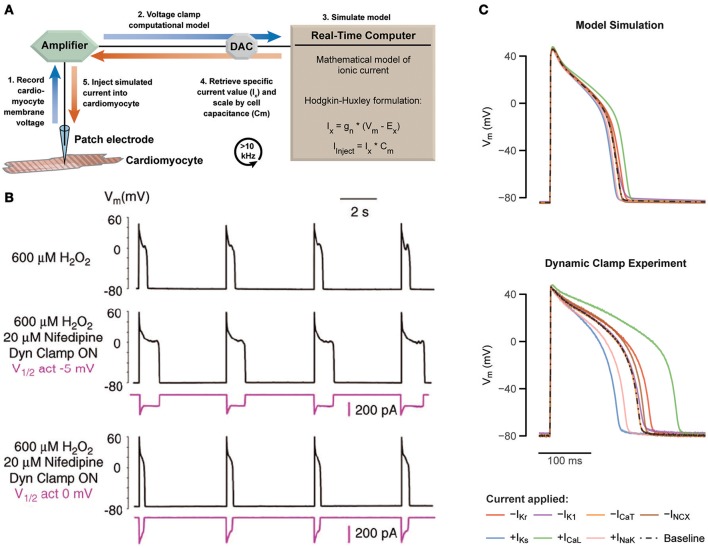
Using dynamic patch clamp to reveal drug targets and systematically test computational models. **(A)** Schematic of the dynamic model clamp configuration. **(B)** Rabbit ventricular myocytes produce EADs during hydrogen peroxide exposure at a pacing cycle length of 5 s at 37°C (middle). Replacement of I_CaL_ with a virtual conductance through dynamic clamp after block with nifedipine recapitulates appearance of EADs (middle). By varying the half-maximal of activation by 5 mV, EADs are abolished (bottom). Adapted with permission (Madhvani et al., 2011). **(C)** Prediction of a 40% increase or decrease of different cardiac currents based on a computational model of a ventricular guinea pig cardiomyocyte are tested with dynamic clamp, revealing a substantial mismatch. Adapted with permission (Devenyi et al., 2017).

Traditional patch clamp protocols are typically static and predetermined, such as sequential voltage steps used to study membrane current dependencies. Dynamic clamp is an extension of patch clamp, where measurements from the cell are used to modify a continuously changing experimental protocol in a real-time feedback loop (Robinson and Kawai, 1993; Sharp et al., 1993). Earlier work has shown broad application—coupling of separate cardiomyocytes through an artificial gap junction (Tan and Joyner, 1990; Joyner et al., 1991; Spitzer et al., 1997; Verheijck et al., 1998; Zaniboni et al., 2000; Huelsing et al., 2001), injection of measured current from a transfected cell into a primary isolated myocyte (Berecki et al., 2005, 2006), antrhomorphization of mouse cardiac APs (Ahrens-Nicklas and Christini, 2009; Bot et al., 2012), and more recently in the study of cardiomyocyte coupling to unexcitable cells (McSpadden et al., 2012) and fibroblasts/myofibroblasts (Nguyen et al., 2012; Brown et al., 2016). The history of dynamic clamp has been reviewed in detail elsewhere (Prinz et al., 2004; Wilders, 2006; Ravagli et al., 2016). Here, we focus on a specific configuration of this technique, called the dynamic model clamp (referred hereafter as dynamic clamp), where a mathematically based model of a conductance is injected to the cell in real-time. Characteristically, this mathematical model describes a specific voltage and time-dependent membrane current determined by a set of differential equations. Measured voltage of a cell in a patch clamp configuration is fed into a mathematical model at high rates, from which the calculated current is injected back into the cell (Figure 1A).

Central to the dynamic clamp experimental rig is the software, which acts as the interface between the patch-clamp hardware and mathematical models. Accurate and rapid sampling of the membrane potential and computation of the virtual conductance is required to mimic sufficiently a biological conductance (Bettencourt et al., 2008). These requirements necessitate hard real-time control. In this context, the feedback loop must complete every iteration within a specified time constraint, typically 50–100 μs (10–20 kHz) in cardiomyocyte dynamic clamp experiments, a feat not possible on standard operating systems and software due to technical limitations. The works discussed here predominately use two software platforms—DynaClamp (Berecki et al., 2005, 2006) and the Real-Time eXperimental Interface (RTXI, www.rtxi.org; Ortega et al., 2014; Patel et al., 2017). Both platforms utilize a customized real-time Linux operating system and are freely available.

In this review, we discuss how investigators have used the dynamic clamp technique to test theoretical drug targets, validate and improve existing cardiac mathematical models, and design assays for cardiotoxicity testing.

## Investigation of arrhythmia mechanisms

### Drug target identification

Dynamic clamp studies on the cardiac L-type Ca^2+^ current (I_CaL_) by Madhvani et al. identified arrhythmia mechanisms, which could potentially be targeted by anti-arrhythmic drugs (Madhvani et al., 2011, 2015). The authors specifically focused on the role of I_CaL_ in the formation of early after depolarizations (EADs), i.e., secondary depolarizations during phase 2 and 3 of the AP resulting from a transient failure of AP repolarization. EADs are used as a marker of cardiac arrhythmia due to its propensity to trigger a premature AP and subsequently initiate cardiac arrhythmias, such as Torsades de pointes (TdP) or ventricular fibrillation, which in turn can lead to sudden cardiac death (Cranefield and Aronson, 1991). EADs require an inward current that can overcome and reverse repolarization, which can be fulfilled by I_CaL_, the major inward current during phase 2 and 3 of the AP. Madhvani et al. aimed to investigate the dependence of EADs on the biophysical properties of I_CaL_, but the lack of an assortment of drugs known to finely alter this current makes traditional patch clamp experiments impractical. Thus, to mimic theoretical perturbations to I_CaL_ properties *in vitro* dynamic clamp was used instead.

In rabbit ventricular myocyte exhibiting EADs, induced with either hydrogen peroxide (Figure 1B, top) or hypokalemia, they replaced native I_CaL_ (blocked with nifedipine) with a virtual model-based I_CaL_, which was injected using dynamic clamp (Figure 1B, middle). The consequences of alterations in I_CaL_ biophysical properties were investigated by manipulating the parameters underlying the modeled current. For example, shifting the half-maximal activation voltage by 5 mV abolished EADs and returned AP duration (APD) to normal values (Figure 1B, bottom). Note that H_2_O_2_ affects multiple inward currents in addition to I_CaL_, such as the late sodium current (Xie et al., 2009), but modification of I_CaL_ alone was able to eliminate EADs.

The mechanistic basis for the observed behavior was established in earlier work describing a window current region between −40 and 0 mV (January and Riddle, 1989) where the steady-state activation and inactivation curves overlap. In this region, a fraction of the L-type Ca^2+^ channels are not inactivated and available for possible reactivation and generation of an EAD. A positive shift in the steady-state activation curve reduces this window region and eliminates EADs. In their later work, Madhvani et al. systematically perturbed all I_CaL_ model parameters and measured the consequences to EAD formation, confirming that parameter changes that reduced the window current region (depolarizing shifts to steady-state activation, or hyperpolarizing shifts to steady-state inactivation) were highly effective at EAD prevention (Madhvani et al., 2015). Based on these observations, the authors identified the purine analog Roscovitine, originally developed as an anti-cancer agent, as a promising anti-arrhythmic due to its ability to decrease the window current through a reduction to the late component of I_CaL_. Preliminary work has shown Roscovitine did indeed abolish EADs in myocytes and terminated ventricular tachycardia/fibrillation in whole rat hearts (Karagueuzian et al., 2017), supporting its therapeutic potential. Notably, this work illustrates a new paradigm in the search for new classes of anti-arrhythmic drugs.

Using a similar approach to the I_CaL_ studies, Altomare et al. investigated the human ether-a-go-go related gene (hERG) channel responsible for the rapid portion of the delayed rectifier K^+^ current (I_Kr_) (Altomare et al., 2015). Mutations and drug perturbations to I_Kr_ result in abnormal repolarization, clinically highlighted by long- or short- QT syndrome. The authors examined how I_Kr_ biophysical properties influenced APD and its temporal variability by blocking and subsequently replacing native I_Kr_ in guinea pig ventricular cardiomyocytes using dynamic clamp. The modeled current was shown to recover control AP parameters adequately, which reveals the properties described in the model are sufficient to describe the contribution of I_Kr_ to APD and its stability. The voltage and time dependent properties of I_Kr_ were systematically perturbed, and then compared to control and drug block conditions. This approach allowed a detailed examination of the consequences of each current property in isolation. The study showed both APD and its variability were most sensitive to changes to steady-state inactivation. Alternatively, while steady-state activation had little impact on APD, significant changes to APD variability were observed. This suggests that variability in APD, rather than mean APD, may be more sensitive in detecting I_Kr_-dependent repolarization abnormalities.

Dynamic clamp has also been used successfully in studies of the transient outward K^+^ current (I_to_), where dynamic clamp was used to vary I_to_ conductance in ventricular (Dong et al., 2006, 2010; Nguyen et al., 2015) and atrial cardiomyocytes (Workman et al., 2012). Given the fact existing I_to_ blocking drugs are non-selective (Ridley et al., 2003; Aréchiga-Figueroa et al., 2010), these studies provided important insight into the relationship between I_to_ and the morphology and duration of the AP. Dong et al. sought to understand the impact of I_to_ on the mechanical properties of cardiomyocytes. I_to_ is responsible for the presence of the characteristic phase-1 notch of the AP, and conflicting evidence suggested notch prominence can either increase or decrease I_CaL_, respectively, enhancing or reducing contraction. Canine ventricular epicardial myocytes are characterized by a prominent phase-1 notch, which endocardial myocytes generally lack (Antzelevitch et al., 1991). By swapping I_to_ conductance levels of both cell-types using dynamic clamp, Dong et al. found that endocardial cells in which the small native I_to_ was substituted by a larger epicardial-like I_to_ displayed diminished contractility, and demonstrated that I_to_ acts as a negative regulator of contractility through reduction of I_CaL_ peak magnitude (Dong et al., 2010).

Workman et al. investigated the influence of I_to_ on atrial arrhythmogenesis, a topic which was unclear due to the lack of I_to_ specific drugs (Workman et al., 2012). Reduction of I_to_ through dynamic clamp revealed AP prolongation, and additional β-adrenergic stimulation evoked EADs. I_to_ increase or exposure to the β-blocker atenolol prevented EAD formation. This suggests I_to_ enhancement holds promise in arrhythmia prevention, at least in the atrium. On the other hand, the dynamic clamp study by Nguyen et al. showed that I_to_ enhancement potentiated EADs in rabbit ventricular myocytes with reduced repolarization reserve, i.e., the intrinsic redundancy against excessive APD (Roden, 1998). By affecting the early AP phases, I_to_ augmentation can alter other voltage-dependent repolarization currents, leading to decreased late repolarization reserve and increased EAD formation (Nguyen et al., 2015).

It is important to note that the dynamic clamp technique suffers from a major limitation, i.e., the lack of ion selectivity in the current injection. Given physiological intracellular solutions contain predominantly K^+^, dynamic clamp of I_CaL_ current will be carried mainly by K^+^, and not Ca^2+^. Thus, the simulated conductance—which should be Ca^2+^-dependent *per se*, is unable to trigger secondary intracellular Ca^2+^ release and contraction. In an attempt to compensate for this limitation, Madhvani et al. simulated the intracellular Ca^2+^ transient, which was then fed back into the I_CaL_ model (Madhvani et al., 2011, 2015), whereas Devenyi et al. included ion selectivity in their simulations (Devenyi et al., 2017). While especially true for Ca^2+^ due to its major role as a secondary messenger, caution should be applied when interpreting results of virtual conductance injection, as transient changes in intracellular concentrations can affect ion channel behavior.

### Improvement of cardiac computational models

The Comprehensive *in vitro* Proarrhythmia Assay (CiPA) initiative seeks to introduce a new cardiac drug safety testing paradigm that combines *in vitro* drug effects on multiple ion channels, computational modeling of cardiac currents and AP, and the use of human stem-cell derived cardiomyocytes (Sager et al., 2014; Colatsky et al., 2016). Computational modeling has proven to be a vital tool in cardiac arrhythmia research, and is expected to be instrumental in the future pipeline in drug testing. Confidence in model accuracy is directly tied to dynamic clamp results, as errors in the formulation of the mathematical model used can skew results. However, this limitation can be exploited because only accurate models can fully rescue behavior after drug block.

Ravagli et al. compared two computational models of the hyperpolarization-activated funny current, I_f_ (Ravagli et al., 2016), which plays a major role in the pacemaker activity current of sinoatrial node (SAN) cells. The authors used a dynamic clamp rescue experiment, where ivabradine was used to partially block I_f_ current, and a dynamic clamp injected model current was used to rescue control behavior. They showed one model significantly outperformed the other by restoring spontaneous activity in SAN cells, identifying the more accurate mathematical formulation of their experimental data. Bartolucci et al. used this strategy to validate an optimized formulation of the I_Kr_ current (Bartolucci et al., 2015). The original Luo-Rudy model (Luo and Rudy, 1994), derived from voltage clamp step protocols (Sanguinetti and Jurkiewicz, 1990), fit poorly to their experimentally measured I_Kr_ current data obtained with AP clamp. After optimization to the AP clamp data, their new model strongly diverged from the widely used Luo-Rudy formulation and fully reversed I_Kr_ block during dynamic clamp.

Devenyi et al. used dynamic clamp to artificially scale multiple cardiac currents in guinea pig ventricular myocytes using a single whole cell model (Devenyi et al., 2017). Altogether, this amounted to a rapid and efficient testing of multiple computationally-based hypotheses within the same cell under static conditions. By comparing their experimental results of the current perturbations to the predicted results from the computational model, the authors noted significant discrepancies (Figure 1C). First, the basal APD was shorter, and second, current perturbations in the experiment were generally larger than predicted by the model. The authors then used the new experimental data to reparameterize the model through unbiased fitting with a genetic algorithm, yielding a new model that could recapitulate the experimental data well. Interestingly, while the original model had a large ratio between the slow (I_Ks_) and rapid (I_Kr_) portions of the delayed rectifier K^+^ current, the fitting consistently reversed this ratio. This finding was then verified experimentally, and further *in-silico* investigation into the consequences to cardiac arrhythmia showed I_Ks_ is better able to prevent EADs during increased L-type Ca^2+^ current.

These studies illustrate how dynamic clamp can be used to experimentally validate computational models, which are typically built from heterogenous data sets spanning numerous experiments, under consistent conditions. Thereafter, new data can be used to further refine the models and advance mechanistic understanding.

## Drug safety testing platforms

Dynamic clamp has also been utilized in the development of new assays for assessment of drug proarrhythmic risks. The current regulatory framework used to prevent approval of drugs with the potential to induce TdP is focused on two main areas: the propensity of the drug to block the hERG channel *in vitro*, and whether the drug prolongs the QTc interval of the ECG. Though largely successful at preventing proarrhythmic drugs from entering the market, the approach has been criticized due to its low specificity, as hERG block and QT prolongation do not always carry torsadogenic risk (Sager et al., 2014; Colatsky et al., 2016). Consequently, it is generally agreed that many promising drugs that may have little arrhythmogenic risk have their development terminated due to failing either criteria. As mentioned previously, the CiPA initiative considers human stem-cell derived cardiomyocytes a key component in future drug safety assays (Sager et al., 2014; Colatsky et al., 2016), and dynamic clamp has been used to address key limitations.

Human induced pluripotent stem cell derived cardiomyocytes (hiPSC-CMs) are being used as an alternative to traditional animal models, cell lines, and heterologous expression systems in the study of cardiac electrophysiology mechanisms and drug-induced arrhythmia. Due to the inherent difficulty in obtaining human cardiac tissue for study, hiPSC-CMs may provide an accessible source of human cell lines and includes the additional capacity to produce patient-specific lines. However, as with human embryonic stem cell derived cardiomyocytes, hiPSC-CMs exhibit an immature phenotype. These cells are stereotypically characterized by spontaneous activity, elevated maximum diastolic potentials, low maximum upstroke velocity, and highly variable APD (Hoekstra et al., 2012). A major contributing factor for these issues is hiPSC-CMs lack of the inward rectifying K^+^ current (I_K1_), which plays a major role maintaining a stable resting potential in quiescent cardiomyocytes (Doss et al., 2012). The lack of I_K1_ is a cumulative issue, in that a generally depolarized membrane potential influences other cardiac currents, such as lowering the availability of fast Na^+^ channels due to inactivation, which reduces upstroke velocity.

**Figure 2:**
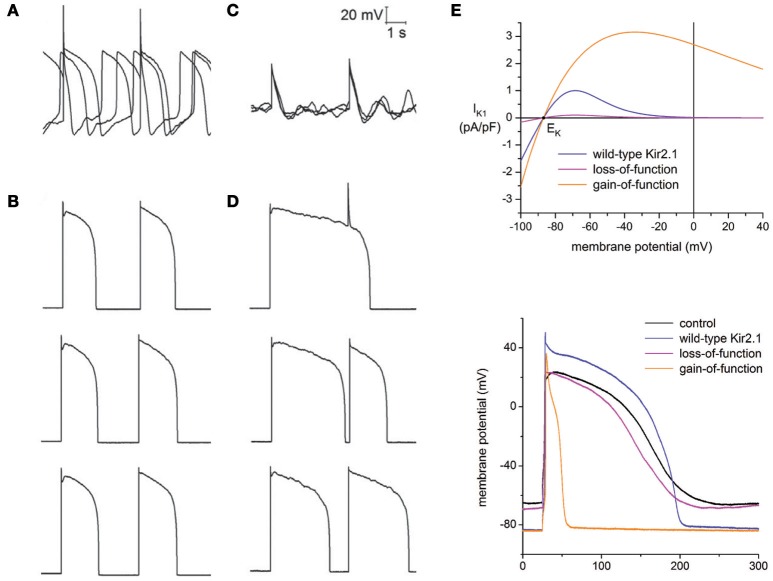
Addressing the immature electrophysiological phenotype of hiPSC-CMs. General lack of the I_K1_ current in hiPSC-CMs plays a major role in their immature phenotype, which was compensated for through I_K1_ dynamic clamp. **(A)** Spontaneous and erratic activity is typical of hiPSC-CMs (average resting potential = −63 ± 5.8 mV, *n* = 21). **(B)** After injection of a virtual I_K1_ current via dynamic clamp, cells become quiescent and produce adult-like stimulated APs (average resting potential = −84 ± 0.1, *n* = 21). **(C)** When exposed to the Ca^2+^ agonist BayK-8644, increased Ca^2+^ loading terminated spontaneous AP generation. **(D)** Exposure of BayK-8644 along with I_K1_ dynamic clamp prolonged APD compared to **(B)**. **(A–D)** adapted with permission (Bett et al., 2013). **(E)** Top panel, current to voltage relationships of I_K1_ models representing wild-type Kir2.1, loss-of-function mutation, and gain-of-function mutation. Corresponding AP morphology during dynamic clamp injection of hiPSC-CMs of each model is shown in the bottom panel. Adapted with permission (Meijer van Putten et al., 2015).

Bett et al. implemented a dynamic clamp based approach to resolve the immaturity issue in hiPSC-CMs through the addition of a virtual I_K1_ current (Bett et al., 2013). The original erratic AP morphology of hiPSC-CMs (Figure 2A) was transformed to an AP profile similar to those seen in adult human cardiomyocytes (Figure 2B), with a stable resting membrane potential and fast upstroke velocity. Seeking to test the impact of the dynamic clamp transformation in response to drug perturbation, hiPSC-CMs were exposed to the Ca^2+^ agonist BayK-8644 at room temperature. Without dynamic clamp, drug addition ceased spontaneous AP generation (Figure 2C) most likely due to BayK-8644 induced Ca^2+^ loading. This is in stark contrast to what is expected from ventricular cardiomyocytes in humans and other mammalian species, where an increase in depolarizing Ca^2+^ currents is expected to increase APD and abnormal activity, such as EADs. With I_K1_ dynamic clamp, however, APD prolongation is evident in stimulated APs (Figure 2D). This illustrates that while hiPSC-CMs are sensitive to BayK-8644, lack of I_K1_ can mask the relevance of drug effects.

Building upon this work, Putten et al. used multiple I_K1_ models in their dynamic clamp experiments to examine the impact of varying degrees of rectification (Meijer van Putten et al., 2015), a biological feature of the I_K1_ current due to differential expression of the channel (Kir2.x) subunits (Wang et al., 1998). Additionally, I_K1_ channelopathies were investigated by modifying their Kir2.1 model to represent gain-of-function and loss-of-function mutations. The gain-of-function mutation was based on the E299V mutation associated with short QT syndrome 3, and the loss-of-function mutation was based on the heterozygous dominant-negative mutation in KCNJ2 associated with Andersen-Tawil syndrome. The top panel of Figure 2E plots the different current-voltage relationships of the modified models. The bottom panel of Figure 2E shows the corresponding APs when these models are used in the calculation of the virtual I_K1_ current during dynamic clamp. Consistent with short QT, the gain-of-function mutation significantly decreased APD, while the loss-of-function had only a marginal effect.

More recently, hiPSC-CM studies augmented with I_K1_ dynamic clamp have provided insight into cardiac abnormalities such as Brugada syndrome (Veerman et al., 2016), long QT syndrome (Rocchetti et al., 2017), and familial atrial fibrilliation (Marczenke et al., 2017). While ion channel dysfunction has been associated with Brugada Syndrome, mainly the cardiac fast Na^+^ current, Veerman et al. found no clear cellular electrophysiological abnormalities in patient-derived hiPSC-CMs, suggesting that other factors, such as fibrosis, could also be underlying mechanisms (Veerman et al., 2016). Rocchetti et al. recently studied hiPSC-CMs derived from a long QT patient carrying a heterozygous mutation in one of the three calmodulin encoding genes (Rocchetti et al., 2017). The patient-specific cells exhibited prolonged APD and failure to shorten with increased pacing rate, which the study linked to impairment of Ca^2+^-dependent inactivation of I_CaL_. The I_CaL_ blocker verapamil reversed mutation-induced repolarization abnormalities. Marczenke et al. explored the role of mutations of the *KCNA5* gene, encoding the channel responsible for the ultrarapid delayed rectifier K^+^ current, in familial atrial fibrillation (Marczenke et al., 2017). The authors generated a functional KCNA5 knockout hiPSC-CM line combining CRISPR/Cas9-mediated mutagenesis and atrial- or ventricular-specific differentiation through manipulation of retinoic acid signaling (Devalla et al., 2015). They observed a strictly atrial-specific disease phenotype, where atrial *KCNA5* knockout hiPSC-CMs exhibited prolonged APD and EADs at low stimulation frequencies vs. insignificant changes in the ventricular variant. These works highlight the potential of hiPSC-CMs in cardiac patient-specific and subtype-specific disease modeling.

I_K1_ dynamic clamp is becoming more common to hiPSC-CM studies to reduce variability in experimental metrics, eliminate spontaneity due to elevated resting membrane potential, and yield a more physiological relevant phenotype. Verkerk et al. systematically analyzed the impact of I_K1_ dynamic clamp on AP characteristics in atrial and ventricular hiPSC-CMs, and provided an in-depth comparison of the methodology and experimental variability of the studies discussed above (Verkerk et al., 2017). While I_K1_ dynamic clamp appears to reduce the variability of most AP parameters, enthusiasm of reducing the large experimental variability of hiPSC-CMs is tempered by the observation that APD variability is not affected. However, elimination of spontaneous depolarizations allows for stimulus at static frequencies, permitting investigation into rate-dependence. More importantly, static pacing reduces beat-to-beat variability, granting a greater ability to detect AP parameter changes. Verkerk et al. also investigated the impact of different mathematical formulations of the injected I_K1_ current, by comparing the models used in several studies discussed previously (Bett et al., 2013; Meijer van Putten et al., 2015; Rocchetti et al., 2017). Not surprisingly, the parameter selection of I_K1_ current density and kinetics can influence relevant AP metrics. Conversely, the flexibility inherent to model modification provides a means to tailor the I_K1_ current to specific cell types, such as ventricular or atrial.

The low throughput of dynamic clamp is a major limitation to its use as part of a drug testing hiPSC-CM platform. Techniques to increase maturation and I_K1_ density, such as 3D culturing (Lemoine et al., 2017) and adenovirus-mediated overexpression of I_K1_ (Vaidyanathan et al., 2016), may circumvent the need for dynamic clamp, but are currently not widely used. Automated patch clamp offers a possible route to increase throughput, but brings a new set of issues, such as interfacing with proprietary equipment and the use of single suspended cells. In a promising recent advance, Goversen et al. have successfully combined I_K1_ dynamic clamp with automated patch clamp of hiPSC-CMs, suggesting the feasibility of high-throughput application as a drug testing platform (Goversen et al., 2017).

In summary, dynamic clamp has been utilized in a number of exciting studies to address some of the inherent limitations of hiPSC-CMs, suggesting a promise as a component of safety pharmacology testing. Furthermore, the ability to modify the underlying mathematical models to examine channelopathies expands the capabilities of this platform.

## Conclusion

By coupling mathematical models with biological experiments, dynamic clamp has provided a powerful tool in the search for potential anti-arrhythmic therapies through model-based perturbations, enhanced hiPSC-CMs as a platform for pharmacological safety testing, and used to clarify and improve mathematical models of cardiac electrophysiology. Dynamic clamp allows fine manipulation of numerous parameters like *in-silico* studies, but is performed in the context of experimental biology. This approach has enabled investigators to test theoretical perturbations in real-time and in live cells, and the power of this technique is represented by the broadness seen in the studies discussed here. It is expected dynamic clamp will continue to elucidate the mechanisms underlying cardiac arrhythmia and identify novel drug targets, and could evolve into a high-throughput assay, e.g., on automated patch clamp platforms to improve maturity of hiPSC-CMs.

## Author contributions

FO, EG, TK-M, and DC all contributed to the planning, writing, and editing of the manuscript and figures contained herein.

### Conflict of interest statement

The authors declare that the research was conducted in the absence of any commercial or financial relationships that could be construed as a potential conflict of interest.
